# HLA-Homozygous iPSC-Derived Mesenchymal Stem Cells Rescue Rotenone-Induced Experimental Leber’s Hereditary Optic Neuropathy-like Models In Vitro and In Vivo

**DOI:** 10.3390/cells12222617

**Published:** 2023-11-13

**Authors:** En-Tung Tsai, Shih-Yuan Peng, You-Ren Wu, Tai-Chi Lin, Chih-Ying Chen, Yu-Hao Liu, Yu-Hsin Tseng, Yu-Jer Hsiao, Huan-Chin Tseng, Wei-Yi Lai, Yi-Ying Lin, Yi-Ping Yang, Shih-Hwa Chiou, Shih-Pin Chen, Yueh Chien

**Affiliations:** 1 Institute of Clinical Medicine, School of Medicine, National Yang Ming Chiao Tung University, Taipei 112201, Taiwan; ettsai@vghtpe.gov.tw (E.-T.T.);; 2 Department of Medical Research, Taipei Veterans General Hospital, Taipei 112201, Taiwan; sypeng0302.12@gmail.com (S.-Y.P.); youren.wu@yahoo.com (Y.-R.W.); tclin6@vghtpe.gov.twenigmaticfog@gmail.com (Y.-H.L.); yj1007hsiao@yahoo.com (Y.-J.H.); a326bcd317@gmail.com (Y.-Y.L.); molly0103@gmail.com (Y.-P.Y.); 3 Institute of Pharmacology, College of Medicine, National Yang Ming Chiao Tung University, Taipei 112304, Taiwan; 4 School of Medicine, College of Medicine, National Yang Ming Chiao Tung University, Taipei 112304, Taiwan; 5 Department of Ophthalmology, Taipei Veterans General Hospital, Taipei 112201, Taiwan; 6 Genomic Research Center, Academia Sinica, Taipei 115024, Taiwan; 7 Department of Neurology, Neurological Institute, Taipei Veterans General Hospital, Taipei 112201, Taiwan

**Keywords:** rotenone, Leber’s hereditary optic neuropathy, mitochondrial complex I, HLA-homozygous iPSCs, mesenchymal stem cells, cell therapy

## Abstract

Background: Mesenchymal stem cells (MSCs) hold promise for cell-based therapy, yet the sourcing, quality, and invasive methods of MSCs impede their mass production and quality control. Induced pluripotent stem cell (iPSC)-derived MSCs (iMSCs) can be infinitely expanded, providing advantages over conventional MSCs in terms of meeting unmet clinical demands. Methods: The potential of MSC therapy for Leber’s hereditary optic neuropathy (LHON) remains uncertain. In this study, we used HLA-homozygous induced pluripotent stem cells to generate iMSCs using a defined protocol, and we examined their therapeutic potential in rotenone-induced LHON-like models in vitro and in vivo. Results: The iMSCs did not cause any tumorigenic incidence or inflammation-related lesions after intravitreal transplantation, and they remained viable for at least nine days in the mouse recipient’s eyes. In addition, iMSCs exhibited significant efficacy in safeguarding retinal ganglion cells (RGCs) from rotenone-induced cytotoxicity in vitro, and they ameliorated CGL+IPL layer thinning and RGC loss in vivo. Optical coherence tomography (OCT) and an electroretinogram demonstrated that iMSCs not only prevented RGC loss and impairments to the retinal architecture, but they also improved retinal electrophysiology performance. Conclusion: The generation of iMSCs via the HLA homozygosity of iPSCs offers a compelling avenue for overcoming the current limitations of MSC-based therapies. The results underscore the potential of iMSCs when addressing retinal disorders, and they highlight their clinical significance, offering renewed hope for individuals affected by LHON and other inherited retinal conditions.

## 1. Introduction

Cell therapy is an emerging therapeutic strategy in translation medicine. Among all cell sources for cell therapy, mesenchymal stem cells (MSCs) have been widely used in various indications. Traditionally, MSCs were considered to have the ability to differentiate between chondrocytes, fibroblasts, and adipocytes [1]. In addition, it has also neem demonstrated that bone marrow MSCs can also produce cardiomyocytes, skeletal muscle cells, and non-mesoderm cells, including neural cells [2,3,4]. Further evidence has indicated that MSCs can secrete neurotrophic factors that promote the survival of nerve cells [5]. Several clinical studies support the use of bone marrow MSCs cells for therapeutic purposes during bone and cartilage repair [6]. The Australian biotechnology company, Cynata, has produced a MSC cell product which has been manufactured to treat osteoarthritis and graft-versus-host disease; currently, it is undergoing separate Phase III and Phase II clinical trials with exciting results and efficacy [7,8]. However, so far, it is necessary to obtain MSCs either from isolated adipose tissue or from bone marrow via invasive procedures. Due to the restricted expansion capability of MSCs, performing these isolation procedures repetitively to obtain a sufficient amount of MSCs is required to meet the demands of cell therapy. Additionally, the high cost associated with in vitro expansion further restricts the clinical accessibility of MSCs, and it hinders their widespread adoption. Traditional methods of MSC acquisition are hindered by the limited proliferative potential of MSCs and donor-to-donor variability, resulting in inconsistent outcomes in clinical trials when compared with MSCs [9,10]. Hence, the identification of an appropriate donor source or the exploration of alternative methodologies for MSC production is imperative to address the existing bottleneck preventing large-scale MSC production.

iPSCs that can be derived from human skin or peripheral blood possess self-renewal and differentiation capabilities that are similar to embryonic stem cells (ESCs), without the accompanying ethical concerns [11,12]. These characteristics allow researchers to utilize iPSCs to generate a variety of lineage cells after defined differentiation, iPSCs highly useful for translational medicine [13,14,15,16,17]. Both iPSCs and ESCs confer the distinct advantage of enabling unlimited MSC production, thus surmounting the constraints associated with donor availability in conventional MSC-based therapeutic approaches. The differentiation capacity of ESCs has been comprehensively investigated, leading to the development of diverse protocols aimed at differentiating between MSCs [18]. Studies in the literature have reported that ESCs can differentiate between MSCs, and they can be applied to conditions such as hypoxic-ischemic brain injuries and models of diseases like multiple sclerosis. It has been noted that MSCs possess immunomodulatory properties and the ability to repair damaged tissues in hypoxic-ischemic brain injuries, as well as multiple sclerosis. [19,20] As with ESCs, iPSCs are also endowed with the capacity to differentiate between MSCs [21]. Numerous studies have also reported that MSCs which differentiate between iPSCs similarly exhibit favorable immunomodulatory effects and tissue-protective capabilities. These effects have been observed in conditions characterized by intense immune response storms, such as osteoarthritis and graft-versus-host disease (GvHD) [22,23]. Remarkably, iPSC-derived mesenchymal stem cells (iMSCs) have been recognized for their ability to meet unmet clinical needs, owing to their inherently inexhaustible characteristics [22]. In contrast to their adult counterparts, iMSCs have demonstrated superiority in terms of cell proliferation, immunomodulatory effects, production of exosomes with microenvironmental regulatory capabilities, and the secretion of bioactive paracrine factors [24]. These findings highlighted the potential utilities of iMSCs over conventional MSCs in translational medicine. However, despite the immense promise of iPSC-based cellular therapy, there exist significant impediments that hinder its practical implementation in the field. Notably, genetic disparities among individuals pose substantial challenges to the widespread adoption of iPSC cellular therapies. Given the distinctive nature of each person’s genome, the utilization of patient-specific iPSCs may necessitate tailored processing and differentiation procedures, thereby augmenting both intricacy and expenditures in the process [9]. Furthermore, these inter-individual genetic variations may exert influence over the stability, therapeutic efficacy, and safety profile of the cells, thereby warranting comprehensive research and evaluation [10]. Consequently, these constraints curtail the scalability and clinical translation of iPSC-based cellular therapy.

Leber’s hereditary optic neuropathy (LHON), an inherited retinal disorder, induces the degeneration of retinal ganglion cells (RGCs), ultimately resulting in bilateral central vision loss and blindness, which is attributed to the malfunction of maternally-inherited, mutated mitochondria. LHON stands out as the most prevalent neuropathy arising from primary mitochondrial mutations, specifically at the m.11778G>A/MT-ND4, m.3460G>A/MT-ND1, and m.14484T>C/MT-ND6 loci within the mitochondrial genes. These loci are collectively responsible for 90–95% of all LHON cases. The prevalence of each mutation is contingent upon the demographic characteristics of the population under investigation. The predominant etiological factor for LHON typically stems from the mutations of the mitochondrial genome (mtDNA), leading to a single amino acid substitution in one of the mtDNA-encoded subunits of the NADH:ubiquinone oxidoreductase, which is the mitochondrial complex I of the electron transport chain. Given the pivotal functions of mitochondrial complex I in mitochondrial operations, any mutations in this complex result in a depletion of energy within neurons, culminating in the demise of retinal ganglion cells (RGCs). Consequently, this phenomenon contributes to blindness in affected individuals [25]. Currently, Idebenone is the only clinically approved drug for the treatment of LHON. As an alternative electron carrier in the electron transport chain, Idebenone can improve cellular ATP production and inhibit lipoperoxide formation [26]. Clinical trials conducted by pharmaceutical companies have shown some improvement in patient outcomes, with a few individuals experiencing vision improvement. However, not all patients respond similarly to the treatment, and the high cost of the medication poses a significant burden to patients. Therefore, there is an urgent clinical need for alternative and more effective LHON treatment strategies.

In an experimental glaucoma model, it was demonstrated that MSCs exhibit neuroprotective efficacy by protecting retinal ganglion cells [27]. Given that LHON is also an inherited retinal disorder characterized by retinal ganglion cell degeneration, the potential neuroprotective effects of MSCs in LHON remain uncertain. Rotenone is reported to be a mitochondrial complex I inhibitor that induces an increase in mitochondrial oxidative stress within retinal ganglion cells, leading to cell death and a reduction in the thickness of the retinal ganglion cell layer (GCL) [28,29,30,31,32]. The rotenone-induced model of complex I dysfunction induces optic neuropathy that is highly similar to LHON. This model is relatively simple to implement, rendering this model attractive for investigating complex I functions. In our previous research paper, we successfully established a highly immune-compatible iPSC line (A33:03-B58:01-DRB1*03:01) (in press). In the current study, we aimed to assess whether this HLA homozygous iPSC line has the capability to differentiate between iPSC-derived mesenchymal stem cells (iMSCs). Additionally, we sought to evaluate the therapeutic potential of iMSCs in experimental rotenone-induced LHON-like models in vitro and in vivo.

## 2. Materials and Methods

### 2.1. Generation of Human iPSCs from the HLA Homozygous Donor

The collection of human peripheral mononucleated cells, and the generation of iPSCs, were approved by the Institutional Review Board of Taipei Veterans General Hospital (Taipei, Taiwan) (TVGH IRB, 2021-04-009A). Human-induced pluripotent stem cells (hiPSCs) were derived from peripheral blood mononuclear cells (PBMCs) obtained from human donors. More specifically, 5 × 10^5^ PBMCs were seeded onto a 24-well plate and cultured in a StemPro^®^-34 medium (Gibco, Gaithersburg, MD, USA), supplemented with 100 ng/mL SCF, 100 ng/mL FLT3, 20 ng/mL IL-3, and 20 ng/mL IL-6 for a duration of four days. Following this, reprogramming was initiated through infection with the Sendai virus, expressing the Yamanaka factors OCT4, SOX2, KLF4, and c-MYC, and utilizing the CytoTune-iPS 2.0 Sendai Reprogramming Kit (Thermo Fisher Scientific, Waltham, MA, USA; A16518) in accordance with the manufacturer’s guidelines. Subsequent to a 24 h incubation post-infection period, PBMCs were harvested, cultured in 1 mL of fresh PBMC complete medium for two days, then transferred to MEF feeders, and sustained in a StemPro-34 medium that was devoid of cytokines. Medium renewal occurred bi-daily. Seven days post-infection, the culture medium was changed to a human embryonic stem cell (hESCs) medium consisting of DMEM/F12 with 20% Knock-out Serum Replacement (KOSR), 1 mM L-glutamine, 0.1 mM non-essential amino acids, 55 µM 2-mercaptoethanol, and 10 ng/mL bFGF, under incubation conditions of 37 °C with a humidified atmosphere of 95% air and 5% CO_2_. Daily medium changes were implemented, leading to the generation and examination of multiple iPSC colonies. To maintain the iPSCs, the StemFlex™ Medium Kit (Thermo Fisher Scientific, Waltham, MA, USA; A3349401) was employed. Culture dishes were pre-coated with a Geltrex™ matrix (Thermo Fisher Scientific, Waltham, MA, USA; A1413301), diluted with cold D-MEM/F-12 (1X) (Thermo Fisher Scientific, Waltham, MA, USA; Cat. no. 10565-018), and incubated in a humidified incubator at 37 °C with 5% CO_2_ for at least 1 h. Sub-culturing was performed using a Versene solution (Thermo Fisher Scientific, Waltham, MA, USA; 15040066).

### 2.2. Alkaline Phosphatase Staining

Alkaline Phosphatase (AP) staining was performed using the Alkaline Phosphatase Kit (Vector Laboratories, Newark, CA, USA), in accordance with the manufacturer’s instructions. Cells were fixed in 80% ethanol for 30 min at room temperature. After fixation, cells were treated with 100 mM Tris-HCl (pH 8.2) for 5 min at room temperature. Then, an alkaline phosphatase working solution (Vector Laboratories, Newark, CA, USA) was added, and the solution was incubated at room temperature for 20–30 min in the dark. Cells were then washed twice with phosphate-buffered saline (PBS). Next, the stained cells were soaked in 100 mM Tris-HCl, and observed under a microscope.

### 2.3. Human iPSC-Derived Mesenchymal Stem Cell Differentiation and Culture

Deriving MSCs from iPSCs was performed in accordance with the protocol described in a previous paper by Kim Hynes et al. [33]. Briefly, human-induced pluripotent stem cells were singled using a Versene solution (Thermo Fisher Scientific, Waltham, MA, USA; 15040066), then, they were centrifuged and re-seeded onto 10 cm dishes pre-coated with a Geltrex matrix (Thermo Fisher Scientific, Waltham, MA, USA; A1413301). The cells were differentiated in a MSC medium, which contains α-MEM medium (Gibco, Carlsbad, CA, USA; Cat. no. 12000063), 2 mM L-glutamine (Gibco, Carlsbad, CA, USA; Cat# 25030081), 100 μM L-ascorbic acid (Sigma-Aldrich, St. Louis, MO, USA; Cat# A4544), 1 mM sodium pyruvate (Gibco, Carlsbad, CA, USA; Cat# 11360070), 50 U/mL Pen Strep (Gibco, Carlsbad, CA, USA; Cat# 15140122), MEM NEAA (Gibco, Carlsbad, CA, USA; Cat# 11140050), HEPES solution (Gibco, Carlsbad, CA, USA; Cat# 15630080), and 10% FBS. This process occurred over 14 days, in a humidified 5% CO_2_ atmosphere, under 37 °C. The medium was changed every 2–3 days as needed. Subsequently, after a 14-day differentiation period, the heterogeneous iMSC population was passaged using TrypLE (Gibco, Carlsbad, CA, USA; Cat# 12563029) to obtain single cells, and it was re-cultured in a MSC medium at a 1:3 ratio. Cells proceeded to subculture once they reached 70–80% density. Cells were defined as passage 1 (P1) after the first passage. From passage 3 onwards, iMSCs were considered fully mature and subjected to the following experiments.

### 2.4. In Vitro Treatment of Rotenone in Cultured Retinal Ganglion Cells

The RGC-5 cell line (ATCC, Manassas, VA, USA) was cultured in Dulbecco’s Modified Eagle’s Medium (DMEM; Gibco, Carlsbad, CA, USA) and supplemented with 10% fetal bovine serum (FBS; Gibco, Carlsbad, CA, USA), 100 U/mL penicillin, and 100 μg/mL streptomycin (Gibco, Carlsbad, CA, USA). Cultivation occurred in a humidified incubator with an atmosphere of 95% air and 5% CO_2_, and the temperature was maintained at 37 °C. The passage of RGC-5 cells was performed when 80% confluence was reached.

Rotenone (Sigma-Aldrich, St. Louis, MO, USA; Cat# R8875) dissolved in DMSO was used to prepare a stock solution for the induction of LHON-like manifestations. To induce LHON in RGC-5 cells in vitro, RGC-5 cells were seeded and cultured at a density of 1 × 10^4^ cells/well, in 300 μL of medium, into 24-well microplates (Corning, Tewksbury, MA, USA). Subsequently, the RGC-5 cells were treated with Rotenone 3 μM for 24 h. After the rotenone treatment, the medium was discarded, and the plates were washed and refilled with serum-free medium. To examine the efficacy of iMSCs, they were seeded onto the Transwell^®^ inserts (Corning, Tewksbury, MA, USA) at a density of 1 × 10^4^ cells/well, and they were co-cultured with RGC-5 cells. After a 48 h co-culture, the iMSCs and the transwell insets were removed.

Cell viability was analyzed using the Cell Counting Kit-8 (CCK8; Beyotime, Shanghai, China), in accordance with the manufacturer’s protocols. After the treatment, 300 μL of serum-free medium, containing 30 μL of CCK-8 reagent, was added to each well, and it was incubated for 2 h. After the incubation, the media were collected, and the absorbance was analyzed at 450 nm, using a microplate reader (Bio-Rad, Hercules, CA, USA) and wells that did not use cells as blanks. The viability of the RGC-5 cells was expressed by the absorbance. All experiments were performed in triplicate.

### 2.5. Measurement of Intracellular Reactive Oxygen Species

To quantify the intracellular reactive oxygen species (ROS) levels in RGC-5 cells, a 2′,7′-dichlorodihydrofluorescin diacetate (DCFH-DA) staining assay (Abcam, Cambridge, UK) was employed. During this procedure, cells were stained by introducing 20 μM DCFDA to the cells in suspension using trypsin. Subsequent to incubation at 37 °C for 30 min, the cells underwent washing with a wash buffer to eliminate excess stains. The stained cells were then subjected to flow cytometry, utilizing 488 laser wavelengths and 535 wavelengths for analysis.

### 2.6. Mitochondrial Reactive Oxygen Species Assay

Mitochondrial reactive oxygen species (ROS) were analyzed utilizing the Mito-SOX™ Red Mitochondrial Superoxide Indicator Kit (Invitrogen, Waltham, MA, USA). The initial step involved diluting the 5 mM MitoSox stock solution from the kit to create a working solution with a concentration of 5 μM MitoSox. The cells earmarked for examination were initially fixed with 4% (*w*/*v*) paraformaldehyde, for 10 min at room temperature, followed by three washes with phosphate-buffered saline (PBS). Subsequently, permeabilization was achieved via treatment with a blocking buffer comprising 5% fetal bovine serum albumin (BSA), 0.3% Triton-X100, and 0.04% Proclin in PBS, for 1 h at room temperature. Post-permeabilization, cells were exposed to a 5 μM MitoSox working solution for 10 min at 37 °C in darkness. Following this, the cells underwent a gentle triple wash with a warm buffer and they were prepared for imaging.

### 2.7. Immunofluorescence Staining 

Cells were cultured in culturing dishes, fixed with 1% (*w*/*v*) paraformaldehyde at room temperature for 10 min. Then, they were washed three times with phosphate-buffered saline, and treated with 5% fetal bovine serum albumin (BSA), 0.3% Triton-X100, and 0.04% Proclin. This was configured in a blocking buffer in phosphate-buffered saline, and the cells were permeabilized at room temperature for 1 h. Next, the antibody that works against the detection protein was added to the cells at a dilution ratio of 1:100, at 4 °C for 16 h. After staining, cells were washed three times with phosphate-buffered saline, and treated with the corresponding secondary antibody (1:500) and DAPI (1:1000) for 1 h at room temperature. Finally, mountant was added, the sample was covered with a coverslip, and it was ready for imaging.

### 2.8. Experimental Animals and Intravitreal Injection of Rotenone

Adult male C57BL/6JNarl mice (6–8 weeks old), sourced from the National Laboratory Animal Center (NLAC, NARLabs, Taipei, Taiwan; https://www.nlac.narl.org.tw (accessed on 2 November 2023)), with an average weight of approximately 18 g, were utilized in this study. All mice adhered to the principles outlined in the ARVO statement regarding the use of animals in ophthalmology and vision research. The mice were housed in a controlled environment at a temperature of 21 °C and humidity set at 55%. They were subjected to a 12 h light and 12 h dark cycle, with ad libitum access to food and water. They received attentive care from trained staff. To assess the tumorigenic risk of iMSCs, adult male ASID mice (6 weeks old) from the NLAC were used.

Rotenone (Sigma-Aldrich, St. Louis, MO, USA; Cat# R8875) was solubilized in DMSO to formulate a stock solution. The injection protocol involved administering rotenone at a concentration of 2 mM, then, it was dissolved in DMSO, and DMSO alone was used as a control. After commencing the rotenone injections, anesthesia was induced in C57BL/6JNarl mice via an intraperitoneal injection of ketamine (80 mg/kg) and xylazine (4 mg/kg). Subsequently, using a 33-gauge needle, 3 μL of the 2 mM rotenone solution was injected into the vitreous of the mouse eye at a 45-degree angle. One day after the rotenone injection, 2 × 10^5^ iMSCs were transplanted into the eyes of the LHON animal model via an intravitreal injection to examine iMSC efficacy on rotenone-injured eyes.

To assess the tumorigenic risk of iMSCs, 1 × 10^6^ iMSCs were subcutaneously injected into the dorsal trunk of ASID mice.

### 2.9. Spectral Domain OCT

Mouse retina detection uses Real-time, Image-Guided OCT2 (Phoenix, Inc; Seeonk, MA, USA). Anesthetized mice were placed on the imaging platform, and the OCT scan of the bottom of the retina of the eye was synchronized with the bright field image, and generated in real time. Image-Guided OCT scans were based on 100 average images of the fundus of both eyes, and they were analyzed for retinal segmentation using InSight software (Phoenix Micron® Inc; InSight Version 2.1.8100; Seeonk, MA, USA). The retina is divided into the following layers: Inner limiting membrane (ILM), Nerve fiber layer (NFL), Ganglion cell layer (GCL), Inner plexiform layer (IPL), Inner nuclear layer (INL), Outer plexiform layer (OPL), Outer nuclear layer (ONL), Outer limiting membrane (OLM), layer of rods and cones (photoreceptors, PR) and the retinal pigmentation epithelium (RPE). The OCT was used to scan the different layers of the retina, including the nerve fiber layer (NFL), ganglion cell layer (GCL), inner plexiform layer (IPL), inner nuclear layer (INL), outer plexiform layer (OPL), outer nuclear layer (ONL), and external limiting membrane. Retinal layer thicknesses were obtained by measuring the four lateral distances (100, 200, 300, and 400 µm) between the optic discs on both sides.

### 2.10. Electroretinography (ERG)

Electroretinography measurements were performed using Celeris (Diagnosys LLC, Lowell, MA, USA). Mice were anesthetized and placed on the Celeris platform. Both eyes were dilated with tropicamide (1%) and phenylephrine (2.5%), and immediately afterwards, hypromellose (2.5%) was applied to the photoconductive electrodes; the mice were placed close to the photoconductive electrodes. The phototransduction electrodes of both eyes were used to perform a 1 Hz flicker ERG frequency at a luminance of 0.5 log cd-s/m^2^, in the frequency range of a-wave rod cell (below 5 Hz), b-wave cone (5–15 Hz), and c-wave cone deviation signals (above 15 Hz). The a-wave amplitude was measured as the difference between the pre-stimulus baseline and a-wave trough, and the a-wave latency was measured as the time taken for the a-wave trough to occur, relative to the stimulus onset. For the b-wave, the amplitude was measured from the a-wave trough to the b-wave peak, and the latency was measured from the stimulus onset to the b-wave peak.

Measuring the ERG pattern was conducted as follows. Briefly, before the ERG pattern tests, the mice were dark-adapted overnight, and the mice’s electroretinograms were measured to test retinal function with a Celeris system (Diagnosys LLC, Lowell, MA, USA). Both eyes were dilated with tropicamide (1%) and phenylephrine (2.5%), and immediately afterwards, hypromellose (2.5%) was applied to the photoconductive electrodes, and the mice were placed close to the photoconductive electrodes. Animals were kept on heating pads to maintain a constant body temperature between 37 and 38 °C for the ERG pattern readings. A drop of gel was placed on the corneal surface. Dark-adapted animals were stimulated with intensities of 50 cd/m^2^ using a stimulator. Recordings of transient PERG responses using alternating black and white strip stimuli were made. The amplitude of P1 was measured from baseline, at a positive peak of P1. The full pattern of the ERG amplitude was measured from the positive peak of P1 to the negative trough of N2.

### 2.11. Statistical Analysis

The data are presented as mean ± standard deviations. Statistical differences between two groups, or among multiple groups, were identified using a paired Student’s two-tailed *t*-test and one-way ANOVA with Tukey’s post hoc analysis, respectively. SPSS Edition 25 software (Chicago, IL, USA) was used for the statistical assessments. A significance threshold of *p* < 0.05 was applied, and highly significant differences were accepted if *p* < 0.001. All presented data are indicative of a minimum of three independent determinations.

## 3. Results

### 3.1. Generation of Human-Induced Pluripotent Stem Cells (hiPSCs)

**Figure 1:**
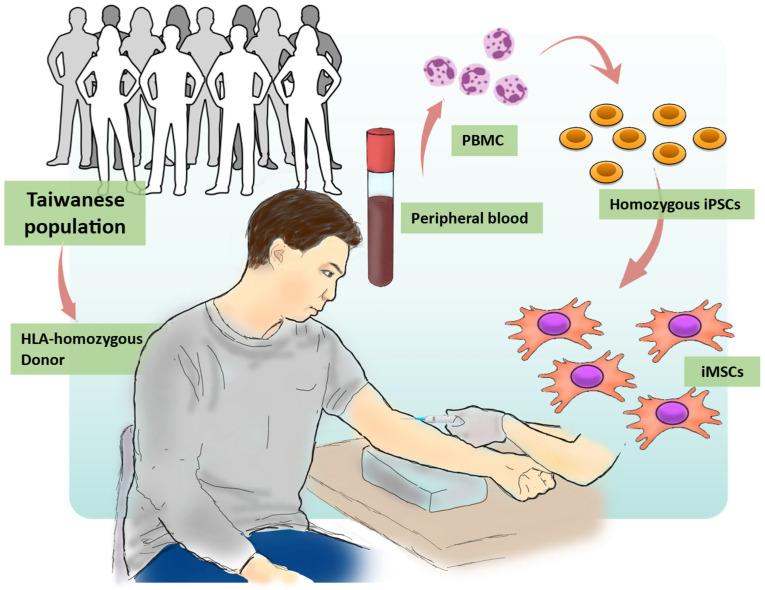
Overview of the process for generating hiPSCs and iMSCs using the HLA homozygous donor. The procedures for iMSC generation in this study involved four steps. (1) HLA Typing: Donors were subjected to HLA analysis to select HLA homozygous donors that match their HLA characteristics. (2) Blood Collection: Peripheral blood mononuclear cells (PBMCs) were collected from the chosen donors. (3) iPSC Generation: Using iPSC technology, PBMCs were reprogrammed to generate iPSCs. (4) Mesenchymal Stem Cell Differentiation: iPSCs were differentiated into iMSCs.

In this study, we recruited a super-donor with an HLA type (A33:03-B58:01-DRB1*03:01; Institutional review board: TVGH IRB, 2021-04-009A) (in press). According to the existing data from the Allele Frequency Net Database (AFND), the A3303-B5801-DRB10301 haplotype is the most prevalent (8.361%) among all reported Taiwanese HLA haplotypes (Table 1). We subsequently isolated the peripheral blood mononucleated cells (PBMCs) from the peripheral blood. These isolated PBMCs were then reprogrammed into human-induced pluripotent stem cells (hiPSCs) using the Cyto-TuneTM-iPS 2.0 Sendai Reprogramming Kit (ThermoFisher, A16518), and Oct4, Sox2, Klf4, and L-Myc were transduced, following previously described protocols [34]. The derived hiPSCs were subsequently differentiated into mesenchymal stem cells (iMSCs), for use in subsequent cell therapy studies (Figure 1).

**Figure 2:**
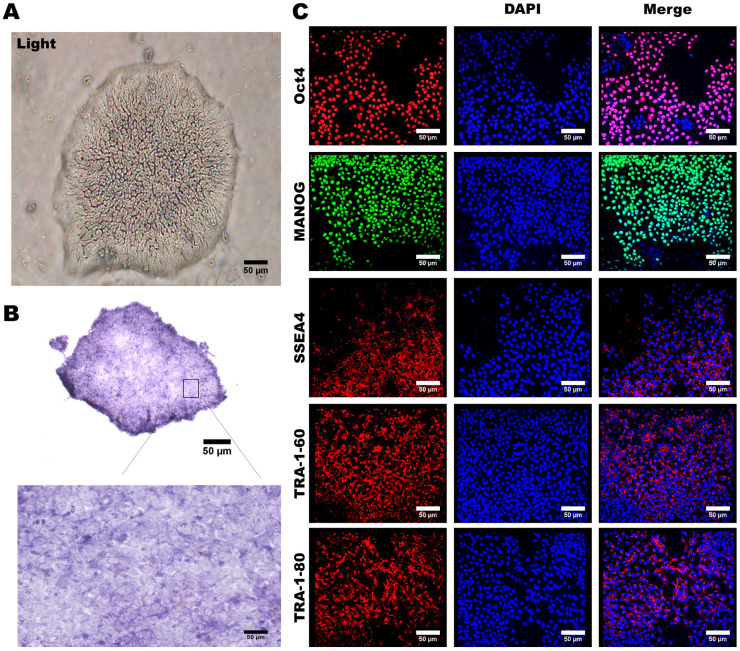
Preparation and Characterization of Human Induced Pluripotent Stem Cells. (**A**) The appearance characteristics of induced pluripotent stem cells, including boundaries and the nucleus-to-cytoplasm ratio, are depicted. (**B**) Strong staining for alkaline phosphatase was observed in induced pluripotent stem cells. (**C**) Immunofluorescence staining revealed the presence of multiple embryonic stem cell-specific biomarkers in induced pluripotent stem cells, including OCT4, NANOG, SOX2, TRA-1-80, and TRA-1-60. Cell nuclei were counterstained in blue using DAPI. Representative images were acquired using a fluorescence microscope with 20× magnification. Scale bar: 50 μm.

Under a microscopic examination, these reprogrammed hiPSCs formed colonies displaying typical characteristics of pluripotent stem cells, including a compact cell arrangement within the colonies, a high nucleus-to-cytoplasm ratio, and distinct cellular margins (Figure 2A). The expression of alkaline phosphatase (ALP) has been widely recognized as a hallmark of ESCs and hiPSCs [35]. To confirm this pluripotent nature, we assessed ALP expression in these HLA homozygous hiPSCs and detected high levels of ALP activity in these hiPSCs (Figure 2B). Furthermore, we conducted immunofluorescence staining to ascertain whether our hiPSCs expressed key pluripotent stem cell markers, including OCT4, NANOG, SOX2, TRA-1-80, and TRA-1-60. Our findings clearly demonstrated that these HLA homozygous hiPSCs are positively stained for these stem cell markers (Figure 2C). Collectively, our data demonstrated that the hiPSCs generated from the HLA homozygous donor exhibit typical characteristics of pluripotent stem cells.

### 3.2. Characterization of MSC-Specific Features in HLA Homozygous iMSCs

**Figure 3:**
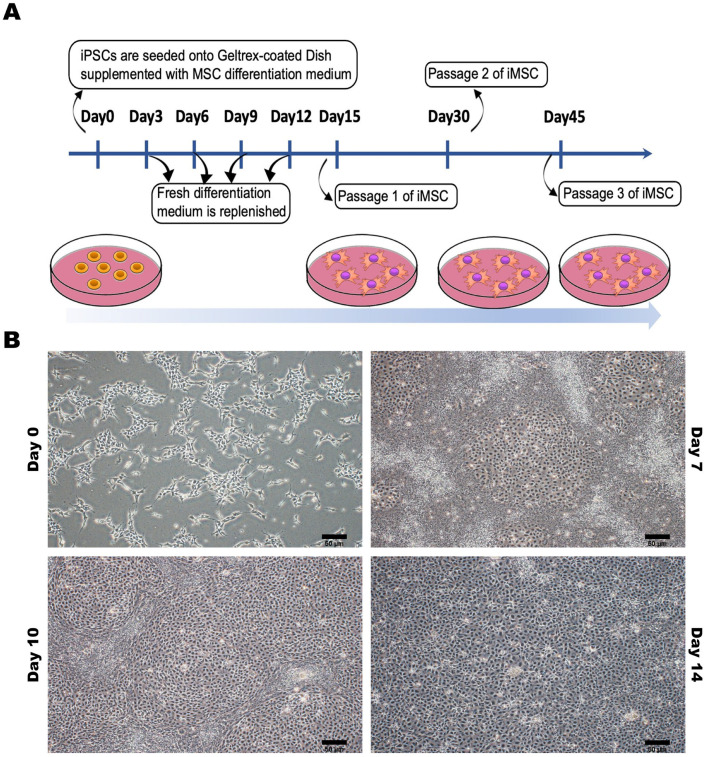
Preparation and Characterization of Human-Induced Pluripotent Stem Cell-Differentiated Mesenchymal Stem Cells (iPSC-MSCs). (**A**) The process of differentiating between human-induced pluripotent stem cells and mesenchymal stem cells. (**B**) The appearance characteristics and morphological changes of human-induced pluripotent stem cells at different time points during the differentiation process into mesenchymal stem cells. Representative images were captured using a microscope with 20× magnification. Scale bar: 50 μm.

To ensure the consistent and uncontroversial production of iMSCs for use as a cell therapy tool, we meticulously established a standardized differentiation process for hiPSCs to differentiate between iMSCs (Figure 3A). This differentiation process involved the induction of an MSC differentiation medium, resulting in observable morphological changes in the hiPSCs within approximately 2 weeks. The cell morphology transitioned from compact structures to a fibroblast-like appearance (Figure 3B).

**Figure 4:**
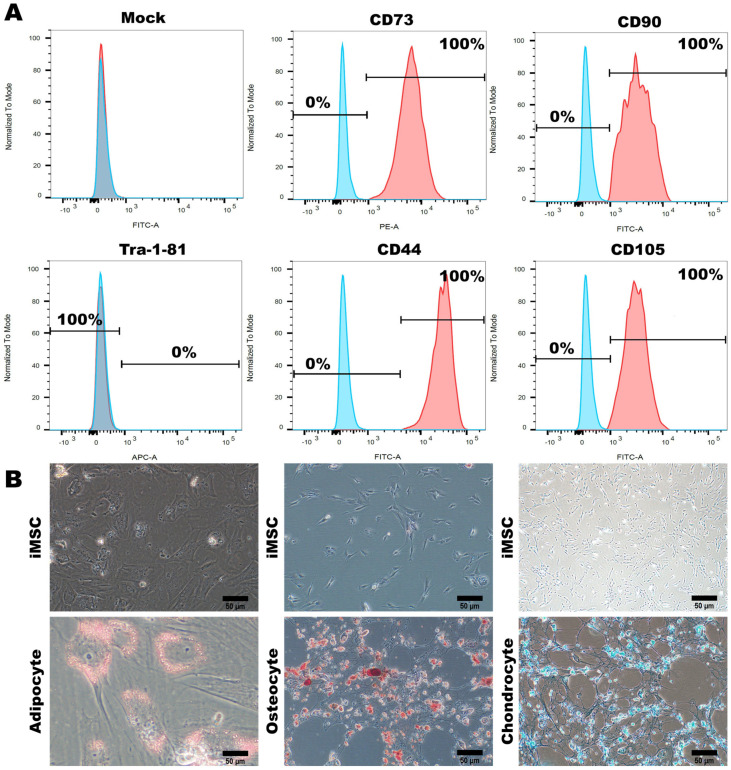
Characterization and Multilineage Differentiation of Human-Induced Pluripotent Stem Cell-Differentiated Mesenchymal Stem Cells (iPSC-MSCs) (**A**) Flow cytometry analysis demonstrated the expression of specific MSC biomarkers (CD73, CD90, CD44, CD105, and Tra-1-81) in hiPSCs before and after differentiating between iMSCs. (**B**) After differentiation occurred, iMSCs displayed the ability to differentiate between adipocytes, chondrocytes, and osteocytes. Representative images were captured using a microscope with 20× magnification. Scale bar: 50 μm.

The resulting population of cells remained at the P0 (passage 0) stage and required continuous culturing and subculturing in an MSC medium to achieve maturation and stability; ultimately, it reached the P3 (third generation) stage. Flow cytometry analysis confirmed the presence of the MSC surface markers CD73, CD90, CD44, and CD105, with a 100% detection rate; however, less than 1% of the cells expressed Tra-1-81 (Figure 4A). Furthermore, we verified the differentiation potential of the iPSC-MSCs that we established, assessing their capability to differentiate between adipocytes, osteocytes, and chondrocytes. This evaluation also served to confirm the maturity and stability of the iPSC-MSCs. Staining with Oil Red O, Alizarin Red, and Alcian Blue revealed the iPSC-MSCs’ ability to differentiate between adipocytes, osteocytes, and chondrocytes, respectively (Figure 4B). These findings validated the diverse characteristics, morphology, and differentiation potential of the MSCs established in our study.

### 3.3. Assessing the Tumorigenic Risk and the Fate of HLA-Homozygous iMSCs in the Xenograft-Bearing Mice

**Figure 5:**
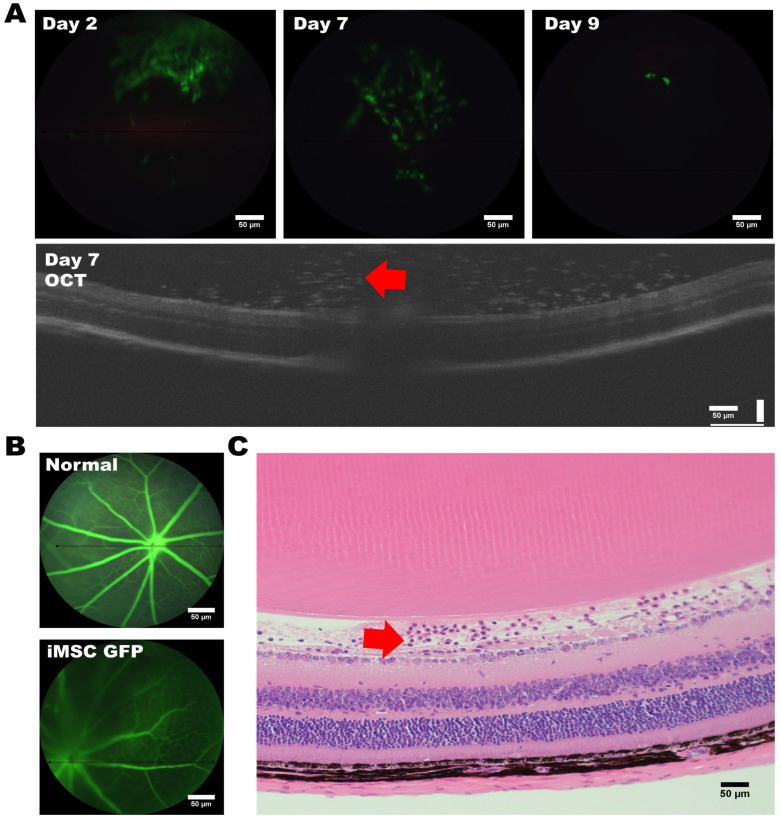
Intravitreal injection of GFP-labeled iMSCs into Mouse Eyes. (**A**) Timeline depicting the intravitreal injection of GFP-labeled iMSCs into the eyes of the mouse recipient, and the iMSCs (indicated by the red arrow) were localized using OCT. (**B**) Comparison of angiogenesis between untreated eyes and eyes with an iMSC transplantation. (**C**) The iMSCs (indicated by the red arrow) were detected in the retinal ganglion cell layer of the retinal structure. Representative images were captured using a microscope with a 20× magnification. Scale bar: 50 μm.

To assess the tumorigenic risk of HLA-homozygous iMSCs, we transplanted 2 × 10^5^ iMSCs into the subcutaneous space of immunocompromised ASID mice. Six months after the transplantation of iMSCs, the histological examination showed that the muscle and skin tissue remained homogenous, and no abnormal hyperplasia formation was found. In order to gain deeper insights into the fate of HLA-homozygous iMSCs following their transplantation into the murine eye, we embarked upon a study to monitor cell survival and examine the adverse effects of iMSC intravitreal transplantation. We utilized HLA-homozygous iPSC-derived MSCs, labeled with a green fluorescent protein (GFP) at a concentration of 2 × 10^5^ cells; they were transplanted via an intravitreal injection into the murine eye. To track the iMSCs’ locations and assess their viability, we employed Phoenix MICRON^®^ Image-Guided Optical Coherence Tomography (OCT). Fluorescent responses were evident through fundus OCT scans as early as the second day post-transplantation; this revealed the presence of a substantial number of iMSCs at the injection site within the vitreous humor (Figure 5A, top left). By the seventh day, we observed the migration of cells towards the optic nerve region at the posterior pole of the eye (Figure 5A, middle), with notable cellular responses proximal to the optic nerve (indicated by red arrows); however, the retinal structure remained unaltered (Figure 5A, OCT). Moreover, from the ninth day onwards, a substantial decline in the fluorescent response of the transplanted MSCs was noted (Figure 5A, top right).

Subsequently, we conducted histological analyses of the transplanted mouse eyes using hematoxylin and eosin (H and E) staining. No inflammation, inflammation-related necrosis, or other abnormalities were noted in the retinal structure. No retinal ganglion cells were affected by the transplantation, and no apparent cell death among the ganglion cell layer (GCL) occurred. Notably, we observed the presence of iMSCs above the retinal ganglion cell layer (Figure 5C, red arrow).

Collectively, our findings suggest that the survival of HLA-homozygous iMSCs, following xenotransplantation into the murine eye, extends for at least nine days. These iMSCs were shown to carry no tumorigenic risk in a long-term follow-up study after transplantation.

### 3.4. iPSC-MSCs Safeguard Retinal Ganglion Cells from Rotenone-Induced Damage In Vitro

**Figure 6:**
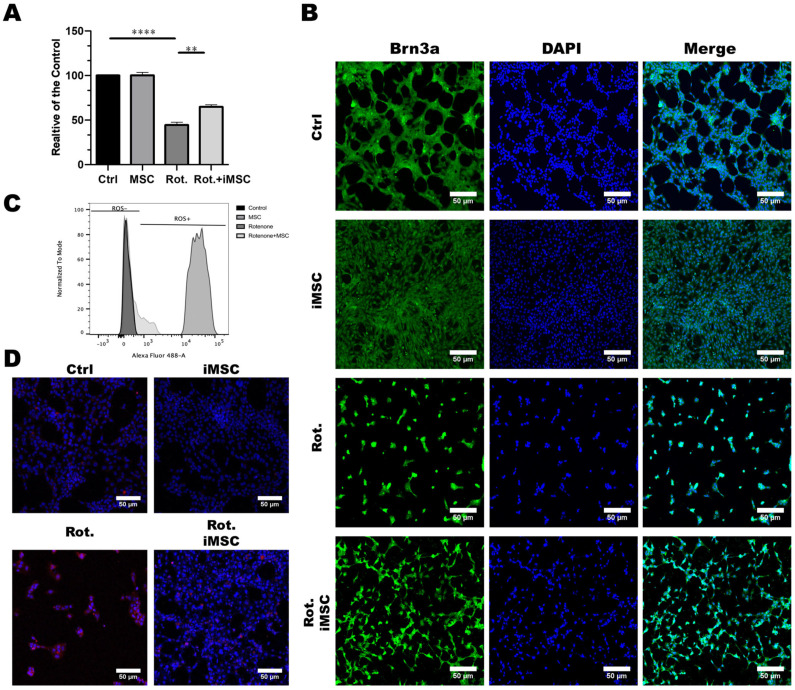
Establishment of the Rotenone-Induced Leber’s Hereditary Optic Neuropathy (LHON) Cell Model and the Effect of iMSCs Treatment. (**A**) RGC-5 cells were pretreated with or without rotenone for 24 h. After discarding the medium, the plates were washed and refilled with a fresh serum-free medium. iMSCs were seeded onto the Transwell^®^ inserts at a density of 1 × 10^4^ cells/well, and they were co-cultured with RGC-5 cells for another 48 h. The toxicity effect of rotenone, and the cytoprotective effect of iMSCs on RGC-5 viability, were evaluated using CCK-8. (**B**) Immunofluorescence staining depicted the expression of the specific surface protein marker, Brn3a, in RGC-5 cells in response to rotenone and iMSC treatment. Cell nuclei were counterstained blue with DAPI. (**C**) Flow cytometry analysis revealed the accumulation of reactive oxygen species (ROS) in RGC-5 cells following exposure to rotenone or iMSC treatment. (**D**) MitoSOX staining illustrated the accumulation of mitochondrial ROS in RGC-5 cells after rotenone or iMSC treatment. Mitochondrial ROS are visualized in red with MitoSox, whereas mitochondria are stained green with MitoTracker. ** *p* < 0.01, **** *p* < 0.0001, were compared between the two groups using a *t*-test. Representative images were captured using a fluorescence microscope at 20× magnification. Scale bar: 50 μm.

To assess the potential therapeutic effects of iMSCs, we first established the rotenone-induced cellular model of LHON in vitro in the RGC-5 retinal ganglion cell line. Considering the toxicity effect of rotenone, which can specifically harm retinal ganglion cells via an increase of oxidative stress, we treated these RGC-5 cells with rotenone for 24 h to simulate the toxicity and damage caused by rotenone to retinal nerve cells. Subsequently, we investigated whether iPSC-MSCs had the potential to improve the rotenone-induced damage to retinal nerve cells. After a 24 h incubation period with rotenone, we completely removed the culture medium, washed the plate, and added fresh serum-free media. To evaluate iMSC efficacy, we seeded iMSCs onto the Transwell^®^ inserts at a density of 1 × 10^4^ cells/well, and we co-cultured them with RGC-5 cells. After being exposed to iMSCs for 48 h, our results revealed that iMSCs did not affect the survival of RGC-5 cells per se (Figure 6A). The preincubation of RGC-5 cells with rotenone led to noticeable cell damage. The co-culturing of iMSCs protected RGC-5 cells from rotenone-induced cell death (Figure 6A).

To further confirm the protective effect of iMSCs, we conducted immunofluorescence staining to observe the expression of the surface protein marker, Brn3a, in RGC-5 cells. The results showed that rotenone treatment led to a significant reduction in the expression of Brn3a in RGC-5 cells, whereas iMSC treatment had a significant protective effect on RGC-5 cells (Figure 6B). It has been reported that the toxic effects of rotenone on cells occurs due to the inhibitory activity of mitochondrial complex I, which generates a large amount of reactive oxygen species (ROS) that ultimately leads to cell death [28]. Therefore, we further investigated whether the protective effect of iPSC-MSCs was related to a reduction in intracellular ROS. We used flow cytometry, in combination with DCFDA/H2DCFDA reagents, to monitor the production of intracellular ROS. The results showed that rotenone-treated mouse retinal ganglion cells accumulated a large amount of ROS inside the cells, whereas the production of ROS in mouse retinal ganglion cells, which were treated with iMSCs, was significantly reduced compared with the rotenone-treated group (Figure 6C). To further validate whether the increase in intracellular ROS after rotenone treatment originated from mitochondria, we used MitoSOX reagents to monitor ROS in mitochondria. The results showed that rotenone-treated retinal ganglion cells exhibited a significant amount of red fluorescence inside mitochondria, indicating the presence of a large amount of ROS; however, this red fluorescence significantly decreased after iMSC treatment (Figure 6D). These results confirm that iMSCs can effectively inhibit the accumulation of intracellular ROS induced by rotenone, thereby protecting cells from rotenone-induced cell death.

### 3.5. Establishment and Validation of the Rotenone-Induced LHON Mouse Model

**Figure 7:**
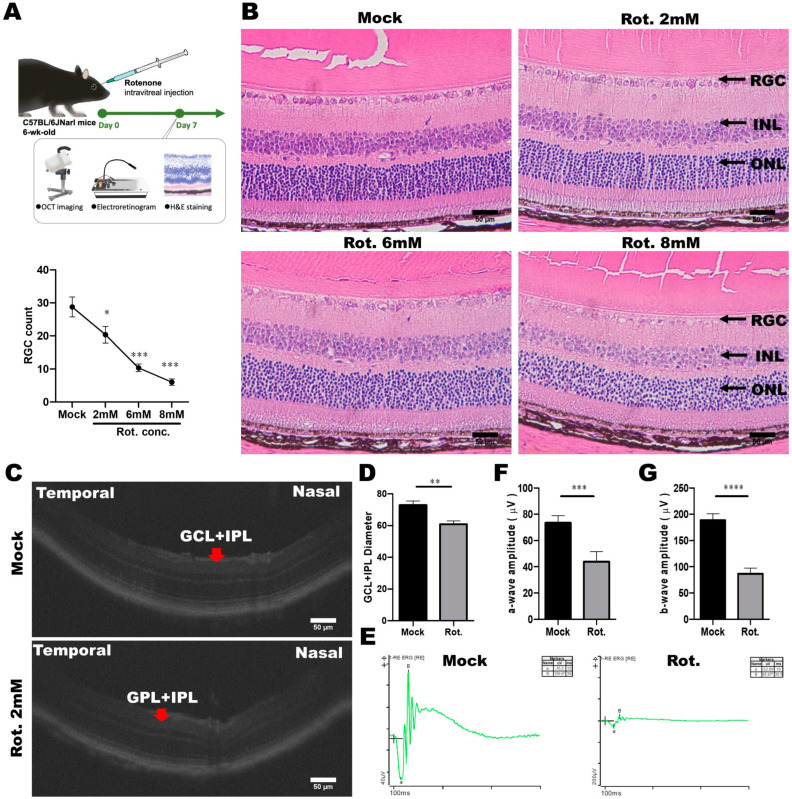
Establishment of a rotenone-induced LHON-like model in vivo. (**A**) Schematic diagram of the animal model of LHON, induced using rotenone, via OCT, ERG, and pathological sections, to verify changes in visual function and structure. (**B**) Effects of different doses of rotenone on retinal ganglion cells in mice. (**C**,**D**) The effect of rotenone on the mouse retina structure (GCL and IPL) was detected using OCT. (**E**–**G**) The effect of rotenone on the electrophysiological a-wave and b-wave of the mouse retina was detected using ERG. * *p* < 0.05, ** *p* < 0.01, *** *p* < 0.001, **** *p* < 0.0001, were compared between two groups using the *t*-test. Representative images were captured using a microscope with 40× magnification. Scale bar: 50 μm.

To explore the potential therapeutic strategies for LHON, we subsequently established an in vivo animal model of LHON induced by rotenone. LHON-like effects were induced through the intravitreal injection of rotenone into the eyes of mice, resulting in the degeneration of retinal ganglion cells (RGCs) within the retina. Subsequently, the mice were euthanized, and their eyeballs were meticulously extracted for further analysis. We employed a comprehensive approach, including optical coherence tomography (OCT), electroretinograms (ERG), and pathological sections, to assess alterations in visual function and structural changes within the eye, as depicted in Figure 7A.

To specifically evaluate the impact of varying rotenone concentrations (2, 4, 6, and 8 mM) on the survival of RGCs, we conducted H and E staining. This analysis revealed a gradual reduction in the number of RGC cells as the rotenone concentration increased (Figure 7B). Notably, rotenone at concentrations of 6 and 8 mM significantly reduced RGC counts to below 10, underscoring its efficacy in inducing RGC cell death (Figure 7B). It is noteworthy that under various doses of rotenone treatment, apart from the GCL layer that is rich in retinal ganglion cells, no significant effects caused by rotenone were observed in the other layers. Our observations unequivocally demonstrated that intravitreal injections of rotenone effectively triggered the demise of retinal ganglion cells in mice.

To further validate the rotenone-induced LHON animal model, we employed Phoenix MICRON^®^ image-guided OCT multifunctional optical tomography to analyze changes in the thickness of the ganglion cell layer (GCL) and inner plexiform layer (IPL) in mouse eyes following rotenone treatment (Figure 7C,D). The results conspicuously revealed a significant thinning of GCL and IPL thickness in the eyes of LHON mice that were treated with rotenone, confirming that the loss of retinal ganglion cells was caused by rotenone treatment.

Moreover, we acknowledged that damaged retinal ganglion cells are closely associated with retinal dysfunction. Consequently, we conducted electrophysiological assessments using the Diagnosys Celeris Electrophysiology System to analyze changes in a-wave and b-wave amplitudes in the eyes of mice treated with rotenone. The a-wave and b-wave correspond with the responses of the photoreceptor and cone/rod system, respectively. Our results revealed a significant reduction in the amplitudes of both the a-wave and b-wave following intravitreal rotenone administration. In mice that were not subjected to rotenone treatment, the flash-induced a-wave exhibited an amplitude of 73.76 μV, and the b-wave exhibited an amplitude of 188.45 μV. However, after rotenone administration, the a-wave amplitude decreased to 43.88 μV, and the b-wave amplitude decreased to 86.3 μV (Figure 7E–G). These data indicated that the rotenone-induced LHON-like model exhibited GCL and IPL thinning, retinal ganglion cell loss, and defective visual signal transduction, without affecting the architecture of inner retinal layers. In summary, we have successfully established a rotenone-induced LHON mouse model, which faithfully replicates LHON-like conditions, and holds promise for further investigations into potential therapeutic interventions.

### 3.6. Evaluating the Treatment Efficacy of iMSC Transplantation in Rotenone-Injured Eyes Using Optical Coherence Tomography and a Histological Examination

As previously mentioned, we utilized the rotenone-induced LHON animal model with a 2 mM rotenone concentration. One day after the induction, we transplanted 2 × 10^5^ iMSCs into the LHON animal model’s eyes via an intravitreal injection. Observation continued until the seventh day, concluding with a retinal structure assessment using the Phoenix MICRON^®^ Image-Guided OCT. Subsequently, the mice were euthanized, and their eyeballs were collected for H and E staining to assess retinal ganglion cell numbers.

**Figure 8:**
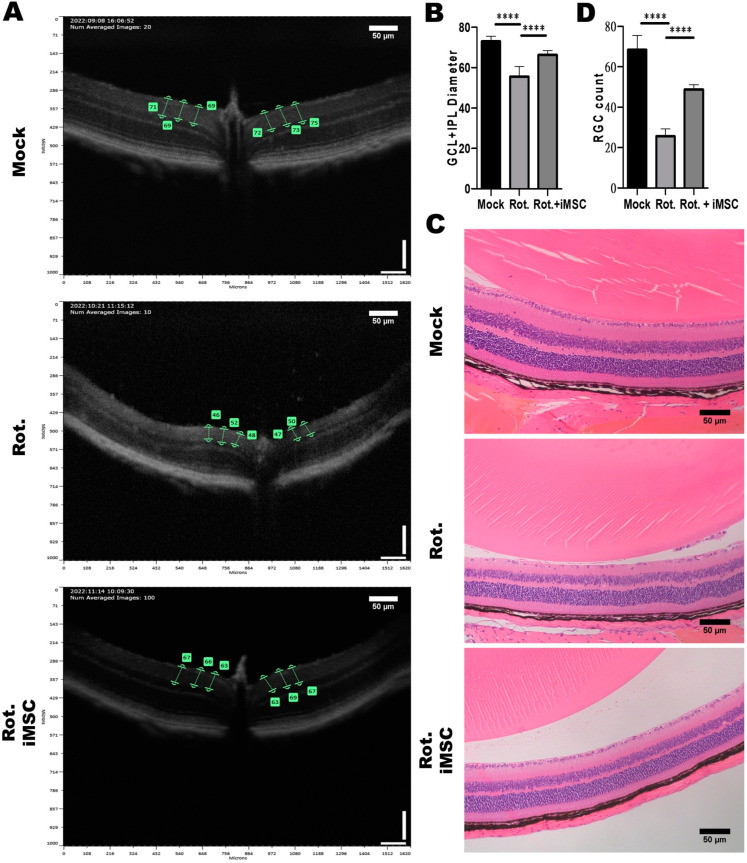
Impact of Intravitreal iMSC transplantation on the Retinal Structure of Mice with Rotenone-Induced LHON-like Optic Neuropathy. (**A**) OCT was used to measure the thicknesses of the retinal layers of mice that were subjected to the abovementioned treatment. The effect of rotenone and iMSC transplantation on GCL and IPL thickness was detected and further quantified, as shown in panel (**B**). (**C**) Histological examination of the retinal architecture of mice that were subjected to the abovementioned treatment. The effect of rotenone and iMSC transplantation on RGC cell count in the GCL layer were assessed and quantified, as shown in panel (**D**). **** *p* < 0.0001, significance assessed via a comparison of the two groups using a *t*-test. Representative images were captured using a microscope at 20× magnification. Scale bar: 50 μm.

The examination of GCL and IPL thicknesses in mouse eyes treated with rotenone revealed significant thinning. Without rotenone treatment, the thickness was approximately 70%, whereas after rotenone treatment, it decreased to about 50% (Figure 8A,B). Similarly, results from the mouse eye sections demonstrated a reduction in the number of retinal ganglion cells to approximately 30 cells post-rotenone treatment (Figure 8C,D).

Following the transplantation of iMSCs into the rotenone-induced LHON-like animal model, we observed an improvement in retinal ganglion cell survival and GCL and IPL thickness 7 days after iMSC transplantation. The thickness increased to approximately 60% (Figure 8A,B), and the H and E results indicated that only a slight reduction in the number of retinal ganglion cells had occurred (to about 50 cells) (Figure 8C,D). These findings indicate that iMSC transplantation into the rotenone-induced LHON animal model can ameliorate the damage inflicted upon retinal ganglion cells by rotenone.

### 3.7. Evaluating the Treatment Efficacy of iMSC Transplantation in Rotenone-Injured Eyes Using the Electroretinogram

To assess the therapeutic potential of iMSCs in the established rotenone-induced LHON-like mouse model, we employed electroretinograms to monitor physiological signals in the rotenone-injured eyes before and after the intravitreal transplantation of iMSCs. The LHON-like animal model was initially induced via an intravitreal injection of rotenone at a concentration of 2 mM, as depicted in Figure 7A. One day later, we transplanted 2 × 10^5^ iMSCs into the rotenone-injured eyes via an intravitreal route. We continued to monitor these animals until the seventh day, at which point, we employed the Diagnosys Celeris Electrophysiology System to measure electrophysiological changes in the mouse eyes.

**Figure 9:**
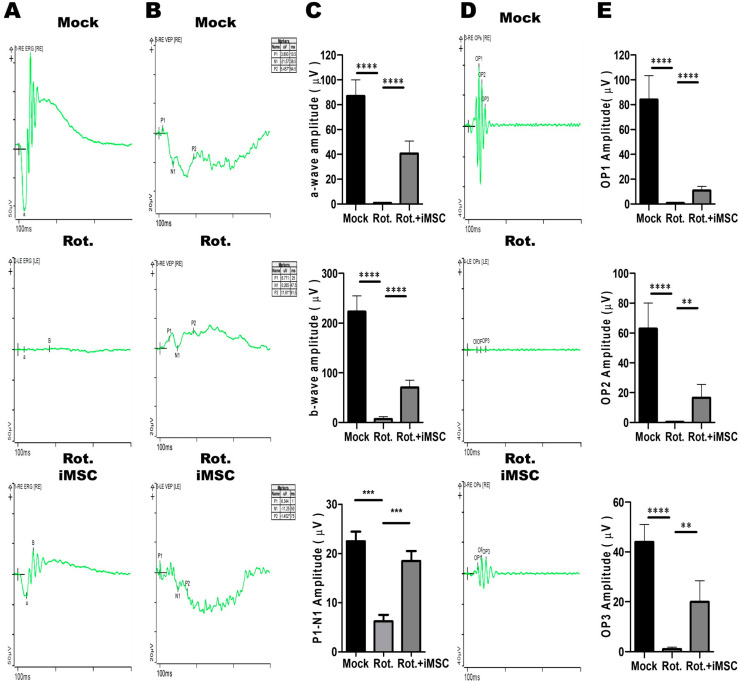
Therapeutic Effects of iMSCs on the retinal electrophysiology in the Rotenone-Induced LHON-like model in vivo. (**A**,**B**) The full-field electroretinogram was used to evaluate the changes in the a-wave, b-wave, and VEP of mouse retinal electrophysiological responses in mice that were subjected to the abovementioned treatment. The changes in the amplitudes of the a-wave, b-wave, and P1-N1 interval were quantified, as shown in panel (**C**). (**D**) The changes in the OP of mouse retinal electrophysiological responses were measured in mice subjected to the abovementioned treatment. The changes in the amplitudes of OP1, OP2, and OP3 were assessed and quantified, as shown in panel (**E**). * *p* < 0.05, ** *p* < 0.01, *** *p* < 0.001, **** *p* < 0.0001 were assessed via a comparison of the two groups using a *t*-test.

Consistent with the observation in our established model (Figure 7), the eyes treated with rotenone exhibited a significant reduction in a-wave (1.354 μV) and b-wave (2.432 μV) amplitudes (Figure 9A,C). Additionally, we measured visually evoked cortical potentials (VEP), which evaluate the electrical signals produced by the visual cortex in response to visual stimulation. The results revealed a significant reduction in P1-N1 amplitudes (6.213 μV) in the eyes treated with rotenone (Figure 9B,C). We also examined oscillatory potentials (OPs), which represent the function of amacrine cells. These measurements showed a reduction in OPs, with electrophysiological signal values of 0.999, 0.198, and 1.574 μV (Figure 9D,E).

Subsequently, we transplanted iMSCs into the eyes of mice that had been previously treated with rotenone. Seven days later, we observed a significant improvement in retinal electrophysiological functions. Compared with the levels observed after rotenone treatment, the amplitudes of the a-wave, b-wave, and VEP, which were induced by the flash, were significantly higher, measuring at 35.64 μV, 74.61 μV, and 18.48 μV, respectively. Furthermore, the oscillatory potentials (OPs) exhibited an increase, with values of 10.26, 20.58, and 18.92 μV, surpassing the levels observed post-rotenone treatment (Figure 9D,E). Collectively, these findings revealed that the intravitreal transplantation of iMSCs into the injured eyes not only ameliorated the rotenone-induced damage in the retinal architecture, but partially rescued the impaired retinal electrophysiological functions.

### 3.8. iMSC Transplantation Partially Rescued Retinal Ganglion Cell Functions in Rotenone-Injured Eyes

The ERG pattern is a specialized ERG test for examining central retinal functions after receiving a contrast-reversing stimulus. Relying on these properties, the ERG pattern can be used for the non-invasive assessment of retinal ganglion cell functions. To examine the electrophysiological functions of retinal ganglion cells in the rotenone-induced LHON-like model, with or without iMSC transplantation, mice that underwent the abovementioned treatment were subjected to ERG pattern measurements. Before the measurements, mice were dark-adapted and stimulated with intensities of 50 cd/m^2^ using a stimulator.

**Figure 10:**
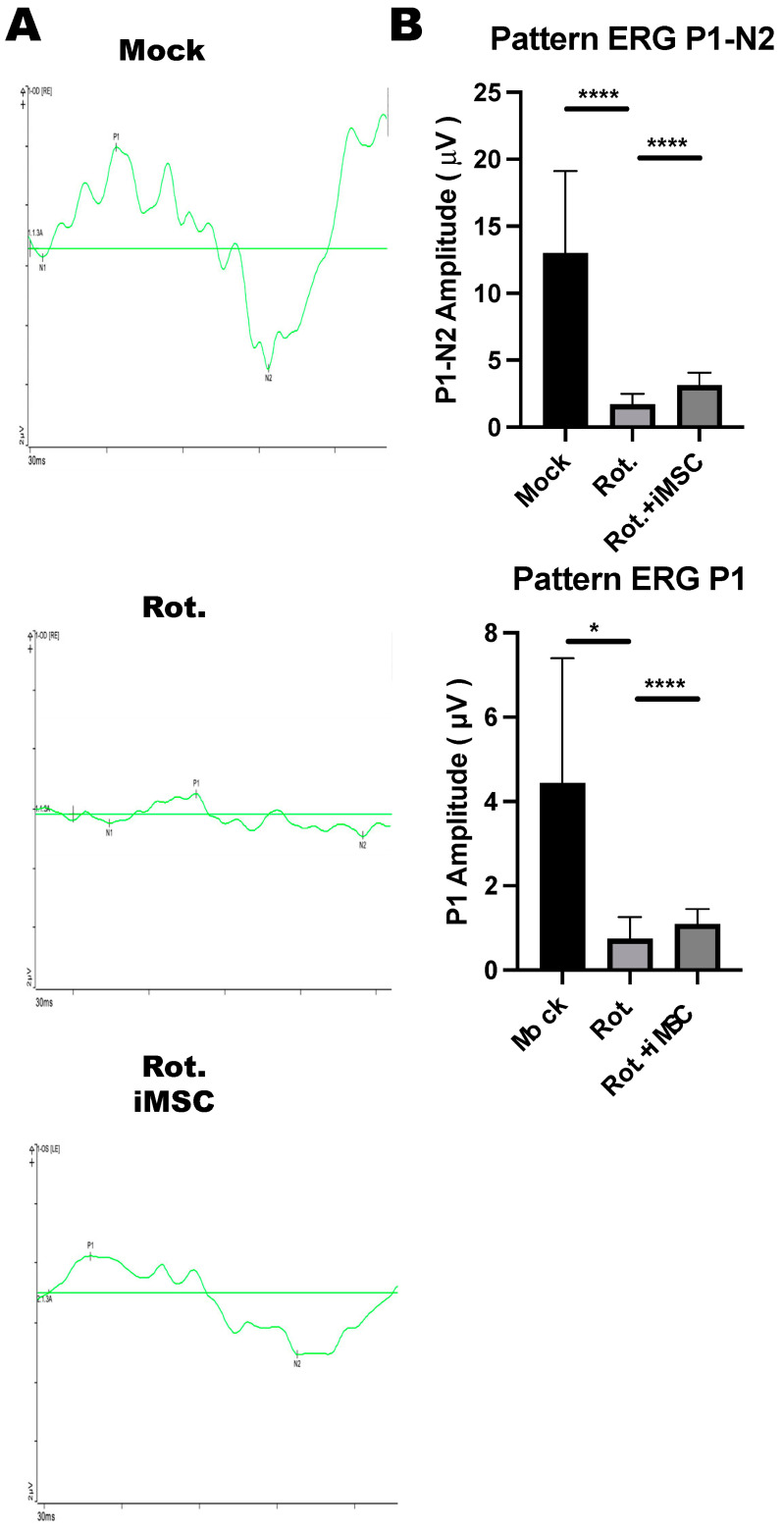
ERG Pattern measurements in the Rotenone-induced LHON-like model. (**A**,**B**) An electroretinogram pattern was used to evaluate the changes in the N1-wave, P1-wave, and N2-wave in mice that were subjected to the abovementioned treatment. The changes in the amplitudes of the P1-wave and P1-N1 interval were quantified, as shown in in panel (**B**). * *p* < 0.05, **** *p* < 0.001 were assessed via a comparison of the two groups using a *t*-test.

The stimulus elicited a remarkable P1 wave, accompanied by a robust decline in the N2 wave, in control untreated mice (Figure 10A,B). The amplitudes for P1 and P1-N2 were reduced in rotenone-injured mice (Figure 10A,B). Notably, the transplantation of iMSCs moderately restored the amplitudes of P1 and P1-N2 in rotenone-treated mice; this reflected the fact that iMSCs partially rescued the functions of retinal ganglion cells that were injured using rotenone.

## 4. Discussion

MSCs play a crucial role in tissue repair and immune regulation, especially for bone and cartilage repair [6], as well as neuroprotection [5], thus demonstrating significant potential for medical applications [7,8]. However, the clinical use of MSCs faces various challenges, including invasive procedures, limited sources, and a decline in effectiveness with advancing age [9,10]. By contrast, iPSCs offer several advantages. They can be derived from adult tissues, they have self-renewal capabilities, and they can differentiate between various cell types without ethical concerns [11,12]. However, iPSC-based therapies encounter challenges related to differentiation control, efficiency, genetic variations, and personalized treatments [36,37].

In order to address the challenges posed by immune system compatibility, investigators have focused on the concept of aligning HLA (human leukocyte antigen) haplotype profiles with donors and recipients, thereby enhancing the compatibility of cellular or tissue grafts with unrelated recipients [38,39]. HLA-matched allogeneic transplantations have the potential to obviate the necessity for immune suppression, and they are safer for the recipients [40,41]. In this study, we successfully obtained human iPSCs (hiPSCs) from the peripheral blood mononuclear cells (PBMCs) of donors with HLA type (A33:03-B58:01-DRB1*03:01), and they differentiated between highly multifunctional iMSCs that exhibited differentiation potential. We investigated the biosafety and fate of HLA-Homozygous iPSC-derived MSCs following transplantation into xenograft-bearing mice eyes. These transplanted cells exhibited early viability and migration towards the optic nerve region within the first week post-transplantation. After the ninth day, there was a noticeable decline in the fluorescence response of the transplanted cells. Histological analysis did not show any inflammation-induced cell necrosis or abnormalities in the retinal structure, and retinal ganglion cells remained unaffected. In summary, the study suggests that HLA-homozygous iMSCs remained viable in the recipient’s murine eye for at least nine days without adversely affecting the retinal ganglion cells or retinal structure. Our long-term follow-up study also showed that these iMSCs did not carry a tumorigenic risk in immunocompromised mice.

LHON is a mitochondrial genetic disease in which the retinal ganglion cell and their axons degenerate, causing acute or chronic blindness or bilateral vision loss. LHON usually occurs mainly in males, and the mutated gene is located in the mitochondrial genome. Since the mitochondria of the embryo come from the ovum, the mutation of LHON is considered to be inherited solely from the mother [25]. LHON is mainly considered to be caused by an abnormality of the enzyme, NADH dehydrogenase (NADH dehydrogenase), found in the mitochondria. The electron transport chain of mitochondria needs a series of protein structures on its inner membrane to assist in the transfer of electrons, and NADH dehydrogenase is the mitochondrial complex I in the electron transport chain [42]. Currently, LHON is commonly thought to be caused by a mutation present from G to A, at position 11,778 on the granulosa chromosome, leading to abnormalities in the MT-ND4 gene [43], of which, 11,778 mutations are very common, accounting for 90% of reported cases in Asian patients and 70% of cases in European patients. Due to an abnormality in the mitochondrial ND4 gene, oxidative stress increases within cells, leading to damage in the retinal ganglion cells [44]. Rotenone is a known substance that disrupts the mitochondrial electron transport chain. It inhibits complex I, thereby disturbing the process of oxidative phosphorylation within cells. This disruption leads to the excessive generation of ROS and subsequent oxidative stress, which damages cellular functions. In LHON, the disease’s onset is closely linked to mitochondrial dysfunction [28]. In the in vitro experiments, our data have demonstrated that under the influence of rotenone, mitochondrial oxidative stress occurred within mouse retinal ganglion cells, leading to cellular damage (Figure 6A–D). Furthermore, by co-culturing iMSCs with the rotenone-injured retinal ganglion cells, we observed a significant increase in the survival of murine retinal ganglion cells when in the presence of iMSCs (Figure 6A,B). The mechanism behind this phenomenon involves the reduction of intracellular mitochondrial oxidative stress, which promotes cell survival (Figure 6C,D). This demonstrates the ability of the HLA homozygous iMSCs to protect retinal ganglion cells.

We also utilized rotenone to establish an LHON-like animal model, aiming to assess the effectiveness of iMSCs as a potential LHON therapy. The reliability of this drug-induced LHON-like model was confirmed by observing a reduction in the number of retinal ganglion cells (RGCs) and a reduction in a-wave and b-wave amplitudes in electroretinograms (ERGs), which are crucial LHON features. These results validate the accurate replication of LHON’s pathological characteristics within the model, providing a robust basis for future therapeutic research. In the rotenone-induced LHON animal model, we observed that in mice, the a-wave represents the functionality of rod and cone cells, whereas the b-wave represents the functionality of bipolar cells, both of which are reduced (Figure 9). This suggests impaired electrophysiology associated with rod, cone, and bipolar cells. Nevertheless, rotenone treatment led to a remarkable amount of retinal ganglion cell death in vitro, and more specifically, it reduced the number of ganglion cells and the thickness of the ganglion cell layer and inner plexiform in vivo, without affecting the architecture of inner retinal layers (Figure 8). Therefore, we attributed the decrease in the a and b waves to the inability of retinal ganglion cells to transmit signals. Through intravitreal injections of iMSCs into LHON animal models, we observed that when retinal ganglion cells are protected, the ability of retinal ganglion cells to transmit signals were effectively restored, and the a and b waves remained responsive. An ERG pattern is a well-understood specialized ERG method which can be used for the non-invasive assessment of retinal ganglion cell functions. Therefore, we further used ERG patterns to clarify the noxious effect of rotenone and the therapeutic potential of iMSCs. The measurements for the ERG patterns indicated that retinal ganglion cell functions deteriorated due to rotenone, but were partially rescued by an iMSC intravitreal transplantation (Figure 10). Furthermore, when examining retinal structure using OCT and H and E staining, our data revealed that the intravitreal transplantation of iMSCs ameliorated GCL and IPL thinning, and it rescued retinal ganglion cells from cell death. Together, we used rotenone to establish in vivo and in vitro models of LHON, successfully replicating key pathological features of the disease. Our results clearly showed that these HLA homozygous iMSCs can protect retinal ganglion cells from rotenone-induced damage, highlighting their great potential as treatment options for LHON.

Numerous investigations have documented the utility of MSCs in diverse ocular pathologies, encompassing age-related macular degeneration [45], Stargardt’s Disease [46], retinitis pigmentosa [47], diabetic retinopathy [48], and glaucoma [49]. Leveraging promising findings from preclinical inquiries, multiple clinical trials were instigated to methodically evaluate MSCs’ prospective advantages in the realm of retinal disease management. iMSCs have been acknowledged for their ability to address unmet clinical requisites, which is attributed to their intrinsically inexhaustible nature [22]. Diverging from adult MSCs, iMSCs have exhibited superiority in cell proliferation, immunomodulatory effects, the generation of exosomes endowed with microenvironmental regulatory capacities, and the release of bioactive paracrine factors [24]. In the present study, our data have elucidated the intravitreal transplantation of iMSCs, which creates potential for protecting retinal ganglion cells from rotenone-induced damage in drug-induced LHON-like murine models. The efficacy of iMSCs was evaluated via OCT, full-field ERG, and ERG patterns. Despite the absence of evidence indicating that iMSCs are capable of rectifying disease-associated genotypes and concurrent ailments in hereditary retinal disorders like LHON, iMSCs demonstrated a noteworthy protective impact on retinal ganglion cells and retinal electrophysiological functions, at least in the acute phase. The mechanisms of iMSCs and their efficacy with regard to retinal ganglion cells may involve the release of neurotrophic factors [50], the initiation of neurogenesis and astroglial activation [51], the promotion of axon growth, augmentation of synaptic connections [52], mitigation of oxidative stress [53,54], and the excretion of exosomes which house a diverse array of bioactive molecules [55,56]. They also have anti-apoptotic and immunomodulatory properties [57,58], as well as anti-inflammatory effects [59] which contribute to a multifaceted spectrum of actions. Prior evidence suggests that, when subjected to suitable stimuli, MSCs exhibit the ability to differentiate between neuronal lineages [60]. This underscores the idea that MSCs might possess the ability to regenerate compromised neural tissue following microenvironmental cues at injury sites. In essence, further preclinical investigations and clinical trials are imperative for comprehensively assessing the long-term and clinical efficacies of iMSCs.

## 5. Conclusions

**Figure 11:**
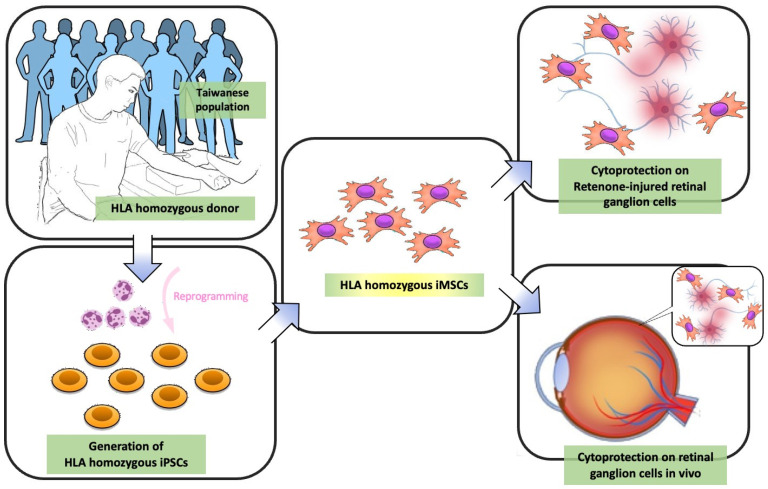
Schematic diagram illustrating LHON model rescue in vitro and in vivo using HLA-homozygous iMSCs. As indicated by the arrows, first, peripheral blood mononucleated cells (PBMCs) were obtained from HLA homozygous donors, and these isolated PBMCs were then reprogrammed into hiPSCs. These hiPSCs subsequently differentiated between iMSCs. This iMSC product holds promise for replacing MSCs in traditional MSC-based therapy, and it may have therapeutic potential in inherited retinal disorders such as LHON.

Despite the significant potential and translational merit of MSCs in cell-based therapy, the insufficiency of cell sources, variable cell qualities, and invasive isolation procedures largely hinder the mass production and quality control of MSCs. The advances in iMSC technologies raise the possibility of solving these problems and enhancing the applicability of MSCs. In the present study, we utilized the highly immune-compatible and HLA homozygous iPSCs, which differentiated between iMSCs using our defined procedures. These generated iMSCs demonstrated ideal potential to protect retinal ganglion cells from rotenone-induced cytotoxicity in vitro and in vivo, at least in the acute phase. This highly immune-compatible iMSC product holds promise for replacing traditional MSCs in cell therapy, and it may have clinical potential for the treatment of inherited retinal disorders such as LHON (Figure 11).

## Figures and Tables

**Table 1:** HLA-A, -B, and -DRB1 allele and allele frequencies (%) in the Taiwanese population.

Rank	Haplotypes	Frequency (%)	Sample Size	Population
1	A*33:03~B*58:01~DRB1*03:01	8.3610	46,628	Taiwan
2	A*02:07~B*46:01~DRB1*09:01	4.4300	46,628	Taiwan
3	A*11:01~B*15:02~DRB1*12:02	1.5000	46,628	Taiwan
4	A*02:03~B*38:02~DRB1*16:02	1.4950	46,628	Taiwan
5	A*30:01~B*13:02~DRB1*07:01	1.3310	46,628	Taiwan
6	A*02:07~B*46:01~DRB1*08:03	1.2620	46,628	Taiwan
7	A*02:01~B*40:01~DRB1*11:01	1.1050	46,628	Taiwan
8	A*11:02~B*27:04~DRB1*12:02	0.9990	46,628	Taiwan
9	A*11:01~B*40:01~DRB1*09:01	0.9290	46,628	Taiwan
10	A*11:01~B*13:01~DRB1*15:01	0.9270	46,628	Taiwan
11	A*11:01~B*46:01~DRB1*09:01	0.8700	46,628	Taiwan
12	A*11:01~B*15:01~DRB1*04:06	0.8110	46,628	Taiwan
13	A*11:01~B*40:01~DRB1*08:03	0.7670	46,628	Taiwan
14	A*11:01~B*40:01~DRB1*11:01	0.7560	46,628	Taiwan
15	A*11:01~B*40:01~DRB1*04:05	0.6770	46,628	Taiwan
16	A*02:03~B*38:02~DRB1*08:03	0.6620	46,628	Taiwan
17	A*11:01~B*13:01~DRB1*16:02	0.6360	46,628	Taiwan
18	A*11:01~B*40:01~DRB1*12:01	0.6340	46,628	Taiwan
19	A*24:02~B*40:01~DRB1*15:01	0.6330	46,628	Taiwan
20	A*24:02~B*40:01~DRB1*09:01	0.5750	46,628	Taiwan

## Data Availability

Data are contained within the article.
